# P-1044. Physician's Compliance to Clinical Practice Guidelines and Outcomes of Patients with Invasive Candidiasis at A University Hospital in Thailand

**DOI:** 10.1093/ofid/ofae631.1234

**Published:** 2025-01-29

**Authors:** Nantaporn Pirogard, Piriyaporn Chongtrakool, Methee Chayakulkeeree

**Affiliations:** Faculty of Medicine Siriraj Hospital, Mahidol University, Bangkok, Krung Thep, Thailand; Faculty of Medicine Siriraj Hospital, Mahidol University, Bangkok, Krung Thep, Thailand; Faculty of Medicine Siriraj Hospital, Mahidol University, Bangkok, Krung Thep, Thailand

## Abstract

**Background:**

Compliance to the clinical practice guidelines (CPG) has shown to reduce mortality in patients with invasive candidiasis (IC). This study aimed to evaluate the physician’s compliance to locally developed CPG after implementation of care bundles and patient’s outcomes.

**Methods:**

This is a quasi-experimental study using historical cohort control. Candidemia patients admitted during November 2021 to April 2024 were enrolled. The CPG for IC was implemented in the prospective cohort. Care bundles were implemented. Each eight CPG item was given a value for poor to good compliance ranging from 0 to 2, and the compliance score was calculated. Compliance score < 8 was considered as poor compliance. Physician’s compliance and all cause 30-day mortality were analyzed.

**Results:**

A total of 109 patients were included, 56 in historical control and 53 in prospective intervention group. Patients in both groups showed similar baseline characteristics and risk factors for candidemia. After the implementation of the CPG and care bundles, the physician’s compliance increased significantly, including early treatment within 24 hours (OR = 5.63, 95% CI [2.25-14.01], *p-*value < 0.001), receipt of appropriate antifungal therapy (OR = 8.67, 95% CI [1.05-71.88], *p*-value 0.03), source control within 48 hours (OR = 34.70, 95% CI [4.36-276.10], *p-*value < 0.001), obtaining blood culture every other day (OR = 17.93, 95% CI [6.90-46.82], *p-*value < 0.001), antifungal therapy at least 14 days after negative blood culture (OR = 2.76, 95% CI [1.20-6.33], *p-*value 0.02), echocardiography (0% vs 17%, *p-*value 0.001), and fundoscopy (OR = 5.25, 95% CI [1.78-15.45], *p-*value 0.001). There was significant improvement in compliance score of ≥ 8 in the intervention group (OR = 5.07, 95% CI [1.86-13.84], *p-*value 0.001). Mean ± SD of the compliance score was 8 ± 2 in the control group and 11 ± 2 in the intervention group (*p*-value 0.001). All cause 30-day mortality rate significantly decreased from 55.4% in the control group to 35.8% in the intervention group (OR = 0.45, 95% CI [0.21-0.97], *p-*value 0.04).

**Conclusion:**

CPG and care bundles for IC significantly improved physician’s compliance and patient’s survival, encouraging the implementation of the CPG and care bundles for the management of IC.

**Disclosures:**

**All Authors**: No reported disclosures

